# Genetic Variants within *NOGGIN*, *COL1A1, COL5A1,* and *IGF2* are Associated with Musculoskeletal Injuries in Elite Male Australian Football League Players: A Preliminary Study

**DOI:** 10.1186/s40798-022-00522-y

**Published:** 2022-10-11

**Authors:** Ysabel Jacob, Ryan S. Anderton, Jodie L. Cochrane Wilkie, Brent Rogalski, Simon M. Laws, Anthony Jones, Tania Spiteri, Dana Hince, Nicolas H. Hart

**Affiliations:** 1 School of Medical and Health Sciences, Edith Cowan University, Perth, WA Australia; 2 Institute for Health Research, University of Notre Dame Australia, Perth, WA Australia; 3 School of Health Science, University of Notre Dame Australia, Perth, WA Australia; 4 Exercise Medicine Research Institute, Edith Cowan University, WA Perth, Australia; 5 West Coast Eagles Football Club, Perth, WA Australia; 6 Centre for Precision Health, Edith Cowan University, Perth, WA Australia; 7 Collaborative Genomics and Translation Group, School of Medical and Health Sciences, Edith Cowan University, Perth, WA Australia; 8 School of Pharmacy and Biomedical Sciences, Faculty of Health Sciences, Curtin Health Innovation Research Institute, Curtin University, Perth, WA Australia; 9 Caring Futures Institute, College of Nursing and Health Sciences, Flinders University, Adelaide, SA Australia; 10 Faculty of Health, School of Nursing, Queensland University of Technology, Brisbane, QLD Australia

**Keywords:** Injury, Muscle, Tendon, Ligament, Bone, Contact, Non-contact, Genetics, Genes

## Abstract

**Introduction:**

Australian Football is a dynamic team sport that requires many athletic traits to succeed. Due to this combination of traits, as well as technical skill and physicality, there are many types of injuries that could occur. Injuries are not only a hindrance to the individual player, but to the team as a whole. Many strength and conditioning personnel strive to minimise injuries to players to accomplish team success.

**Purpose:**

To investigate whether selected polymorphisms have an association with injury occurrence in elite male Australian Football players.

**Methods:**

Using DNA obtained from 46 elite male players, we investigated the associations of injury-related polymorphisms across multiple genes (*ACTN3, CCL2, COL1A1, COL5A1, COL12A1, EMILIN1, IGF2, NOGGIN, SMAD6*) with injury incidence, severity, type (contact and non-contact), and tissue (muscle, bone, tendon, ligament) over 7 years in one Australian Football League team.

**Results:**

A significant association was observed between the rs1372857 variant in *NOGGIN* (*p* = 0.023) and the number of total muscle injuries, with carriers of the GG genotype having a higher estimated number of injuries, and moderate, or combined moderate and high severity rated total muscle injuries. The *COL5A1* rs12722TT genotype also had a significant association (*p* = 0.028) with the number of total muscle injuries. The *COL5A1* variant also had a significant association with contact bone injuries (*p* = 0.030), with a significant association being found with moderate rated injuries. The *IGF2* rs3213221-CC variant was significantly associated with a higher estimated number of contact tendon injuries per game (*p* = 0.028), while a higher estimated number of total ligament (*p* = 0.019) and non-contact ligament (*p* = *0.002*) injuries per game were significantly associated with carriage of the *COL1A1* rs1800012-TT genotype.

**Conclusions:**

Our preliminary study is the first to examine associations between genetic variants and injury in Australian Football. *NOGGIN* rs1372857-GG, *COL5A1* rs12722-TT, *IGF2* rs3213221-CC, and *COL1A1* rs1800012-TT genotypes held various associations with muscle-, bone-, tendon- and ligament-related injuries of differing severities. To further increase our understanding of these, and other, genetic variant associations with injury, competition-wide AFL studies that use more players and a larger array of gene candidates is essential.

## Key Points

- In this select cohort of elite Australian Football players, those with the rs1372857-*NOGGIN* GG or *COL5A1* rs12722-TT genotype had a higher estimated number of total muscle-related injuries per game. However, those with the *NOGGIN* rs1372857-GG genotype also had a higher estimated number of moderate-to-high severity rated injuries.
- The *IGF2* rs3213221-CC genotype had a higher estimated number of contact tendon injuries per game, and a higher number of low severity rated injuries.
- The rs1800012-TT genotype of *COL1A1* had a higher number of ligament-related injuries per game, with significant associations seen between the genotype and low severity rated injuries.
- The *COL5A1* rs12722-TT genotype had a higher number of contact bone injuries per game, and a higher number of moderate severity rated injuries.

## Introduction

Australian Football (AF) is a unique endurance-based team sport interspersed with many high-intensity efforts across a match [1–4]. In the elite competition, the Australian Football League (AFL), players regularly run over 13-kms per match over a 120-min period [5]. Players continuously perform at an intense physical level due to the dynamic nature of the sport which requires players to accelerate and decelerate, change direction, and explosively jump repeatedly, while also performing sport specific skills such as kicking, handballing, marking, and tackling [6–10]. Due to the demands of the sport, injuries occur frequently with the most common injuries including hamstring strains, anterior cruciate ligament (ACL) ruptures, glenohumeral dislocations, leg and foot fractures (i.e. mainly stress fractures), and ankle joint injuries [11]. To be successful, AFL teams not only need talented athletes, but also lower injury rates to optimise player availability for team selection [12], as greater team continuity allows less disruption of athlete personnel during a season, which can lead to better team consistency and success [12]. Despite concerted efforts from strength and conditioning coaches, and medical staff, there is still an unknown combination of extrinsic and intrinsic mechanisms that may affect an athlete’s rate of injury and rate of recovery from injury, including genetic factors [13–15].

The expression of certain genes influences various physical athletic qualities such as body composition, muscle stiffness, elasticity and strength, and response to exercise-induced muscle damage [16–18]. Single nucleotide polymorphisms (SNPs) are naturally occurring variances in the human genome at a specific position, where an individual may have a pair of the same DNA bases (homozygous) or two different DNA bases (heterozygous) [19]. These polymorphisms could have no effect or could be beneficial or deleterious to the individual [19]. SNPs can have an effect on musculoskeletal formation, structure, repair, blood flow and metabolism [20–22], influencing muscles, tendons, and ligaments [23]. The presence of SNPs in different genes has been shown to be related to musculoskeletal injury risk (alpha-actinin-3 [*ACTN3*] [24]; collagen type I alpha 1 [*COL1A1*] [25]), musculoskeletal injury occurrence (collagen type V alpha 1 [*COL5A1*]) [14]), muscle injury severity (*COL5A1*; insulin-like growth factor-2 [*IGF2*]; chemokine CC motif ligand-2 [*CCL2*]) [26]), muscular strength (*CCL2* [23]), an influence on muscle function (COL5A1 [27]), increased risk of ACL injury (*COL1A1*; and collagen type XII alpha 1 [*COL12A1*] [28, 29]), ligament injury severity (elastin microfibril interface 1 [*EMILIN1*] [26]), bone mineral density (*NOGGIN*; and SMAD Family Member 6 [*SMAD6*] [30–32]), and injury recovery time (*IGF2*; *CCL2* [26]).

SNPs in the *COL1A1*, *COL5A1*, and *COL12A1* genes have all been reported to affect the production of their associated collagen types. *COL1A1* affects cell adhesion and differentiation [33], *COL5A1* is a part of the extracellular matrix and can influence the production of type V collagen by altering mRNA stability [34], and *COL12A1* is the link between fibrils and its mutations [35]. *ACTN3* influences skeletal muscle formation as it forms the part of the fibres responsible for rapid and forceful contractions [36]. *IGF2* regulates cell proliferation, growth, migration, differentiation, and survival via its protein hormone [37]. *CCL2* can recruit monocytes, memory T cells, and dendritic cells as part of the CC chemokine superfamily responsible for chemotactic activity and increases in calcium influx [38]. *EMILIN1* regulates systemic blood pressure, as well as being part of the extracellular matrix [39–41]. *NOGGIN* is an extracellular antagonist of the bone morphogenetic proteins (BMPs) that regulate heart development via complex morphogenetic processes [42]. *SMAD6* affects its protein which is an intracellular mediator of signalling caused by BMPs [43].

Genetic variants in multiple genes have been associated with injuries in clinical [25, 44, 45] and athletic [44, 46–48] populations. Within AF, team success relies on many factors such as talent, skill, and fitness; however, lower injury rates are also important, as injuries can keep players from playing for a period of time or to the best of their ability, and therefore, understanding potential links to injury, such as genetics, is important. Research has focussed on mechanisms of injury, for example, type of technique, loading, muscle strength, timing of muscle activation, previous injury history, age and fatigue [49–51], and external factors, such as ground contact forces and ground–shoe friction [11, 49], causing injury in AF. However, the association between genetic variants and injuries in AF has not been investigated. Accordingly, the purpose of this study is to investigate whether the previously researched candidate polymorphism has an association with injury occurrence in an elite AFL playing squad. We hypothesise that there will be a genetic influence on injury occurrence for at least one of the candidate polymorphisms.

## Materials and Methods

### Study Design

A prospective longitudinal cohort study was conducted across seven consecutive seasons (2011 to 2017) used to investigate the association of injury-related SNPs, in nine genes (*ACTN3, CCL2, COL1A1, COL5A1, COL12A1, EMILIN1, IGF2, NOGGIN, and SMAD6)*, in a population of professional, elite AF players to explore possible relationships between candidate SNPs and injury outcomes (incidence [total, contact, non-contact] and severity [low, moderate, high]) relating to muscle, tendon, ligament, and bone. Estimated numbers of injuries per genotype for each genetic variant are reported to provide easy-to-interpret presentation of data for use by strength and conditioning coaches.

### Participants

Forty-six (n = 46) elite male AF players were recruited from one AFL team to participate in the study, as previously described [52, 53]. For each in-season round, 22 to 23 players were selected to play in the AFL competition, with the remaining 24 to 25 playing in the state competition for that given round (WAFL; Western Australian Football League). Anonymity was ensured by assigning players with a randomised, non-identifiable code. All participants provided written informed consent after being provided with information letters outlining the purpose of the study and potential benefits and risks. All data collection and management procedures conformed to the Declaration of Helsinki (World Medical Association) with ethics approved by the Edith Cowan University Human Research and Ethics Committee (ID: 2019-00181-JACOB).

### Injury Data Collection

Data were collected from medical and injury reports provided by the AFL club, recorded by their medical professionals, including doctors, physiotherapists, and strength and conditioning personnel. Injuries were diagnosed with a description of location and categorised as non-contact (i.e. intrinsic injuries that are acute, chronic, or overuse injuries stemming from the athlete themselves) or contact (i.e. extrinsic injuries that are acute collision or contact injuries stemming from external forces). Club medical staff provided each injury with a severity rating that is based on the number of training sessions and games that elapsed from the date of injury to the date of the player's return to full participation in team training with availability for match selection. Specifically, severity was graded as low (i.e. training is modified or less than 1 week of training is missed, with no games missed), moderate (i.e. 1–2 weeks of training missed, or unavailable for 1 to 2 games), or high (i.e. 3 or more weeks of training missed, or unavailable for 3 or more games) as an internal club-determined metric. All injuries were then placed into a sub-category: bursitis; concussion; contusion/bruise/haematoma; dislocation/subluxation; fracture; lesion of meniscus, cartilage or disc; muscle strain/tear/rupture/cramps; other bone injuries; other injuries; sprain/ligament injury; joint instability; tendinopathy; or tendon injury/rupture. In our study, injuries that could be categorised into the broad categories of (1) muscle (including muscle strain/tear/rupture/cramps), (2) tendon (tendinopathy, and tendon injury/rupture), (3) ligament (including sprain/ligament injury, and joint instability), and (4) bone (including fracture, and other bone injuries) were used. Data were collected over 7 consecutive seasons (2011 to 2017). Injuries occurring in training sessions or matches during the pre-season (between 17 and 21 weeks depending on the year and season over the Australian summer) and in-season (between 23 and 27 weeks depending on the year and season over the Australian winter with the inclusion of a final series when applicable) were included. Players who were injury-free in-season were either selected to play in the national AFL competition or played in the state WAFL competition. Due to the professional nature of the team and club, all players undertook a similar volume of training when not injured.

### Sample Collection and DNA Analysis

Buccal saliva samples were collected via mouth swabs with participants instructed to brush the edge of a soft tip swab along the insides of their cheek and gums for 30 s [52–54]. Samples were collected before a pre-season training session and players were asked not to consume coffee, alcohol, or food for two hours prior to saliva collection. Collected samples were labelled with a numeric code for de-identification and were sent to the Australian Genome Research Facility (AGRF; Brisbane, QLD, Australia; NATA 17025) for DNA extraction and genotyping using Agena Bioscience MassARRAY system (AGRF; Brisbane, QLD, Australia). Genetic variants examined were within the following genes: *ACTN3* (rs1815739), *CCL2* (rs2857656)*, COL1A1* (rs1800012)*, COL5A1* (rs12722)*, COL12A1* (rs970547)*, EMILIN1* (rs2289360)*, IGF2* (rs3213221)*, NOGGIN* (rs1372857)*, SMAD6* (rs2053423), with all genotypes being within Hardy–Weinberg equilibrium (HWE), as previously reported [52].

### Statistical Analysis

Data were statistically analysed using IBM-SPSS V.24 (Armonk, NY, USA) and Stata/BE v17 (StataCorp LLC, Collage Station, TX, USA). A negative binomial distribution model was used to assess the relationship between the total number of injuries per injury category (muscle, tendon, ligament, and bone) and the genotypes of each of the candidate genes. Each injury category was analysed in total and via non-contact (i.e. intrinsic), and contact (i.e. extrinsic) injury mechanism. The number of games played was included as the offset variable to account for differences in exposure to the risk of injury. Genotype was considered a continuous variable to test for the linear trend in the association. Significant association with a particular gene was followed up with separate models to determine if there was an association between the number of injuries within each injury category and their severity (low, moderate, high, and a combination group of moderate and high). These results are reported as incident rate ratios (IRRs) and estimated number of injuries with 95% confidence intervals (95% CI). A significant nominal *p* value of < 0.05 was employed.

## Results

Longitudinal team descriptive results are presented in Table 1, with genotype frequencies presented in Table 2. The average number of injuries for each variant per season is presented in Table 3 for muscle, tendon, ligament, and bone-related injuries. Over the seven seasons, 992 incidents of injury were included, of which 351 incidents were contact injuries, and 553 were non-contact injuries, with 88 injuries unclassified by the football club personnel at the point of collection. Within injury categories, (1) muscle injuries had 299 incidents [21 contact, 261 non-contact, 17 unclassified]; (2) tendon injuries had 73 incidents [8 contact, 60 non-contact, 5 unclassified]; (3) ligament injuries had 304 incidents [203 contact, 73 non-contact, 28 unclassified]; and (4) bone injuries had 111 incidents [39 contact, 66 non-contact, 6 unclassified].

**Table 1:** Descriptive statistics and mean player injuries for each season

AFL season							
Variable	Season 1 (n = 46)	Season 2 (n = 40)	Season 3 (n = 29)	Season 4 (n = 25)	Season 5 (n = 21)	Season 6 (n = 18)	Season 7 (n = 15)
Age (Years)	24.94 (4.26)	24.20 (4.02)	23.90 (3.21)	23.60 (2.87)	23.38 (2.42)	22.50 (2.50)	21.93 (2.38)
Active weeks	35.23 (6.25)	35.73 (6.02)	41.45 (5.42)	36.60 (3.43)	35.19 (5.38)	37.28 (5.85)	41.20 (3.92)
Games played	17.45 (45)	18.78 (2.92)	20.03 (6.17)	18.60 (3.43)	17.57 (4.50)	19.28 (5.85)	20.73 (3.97)
Games lost	4.62 (6.72)	1.60 (2.92)	4.31 (6.93)	2.56 (3.56)	4.62 (4.58)	3.83 (6.29)	3.40 (4.38)
All Injuries	1.85 (1.61)	2.14 (3.35)	1.61 (1.37)	1.78 (1.19)	2.20 (1.56)	1.57 (1.16)	1.50 (1.15)
Muscle	3.40 (1.80)	2.08 (1.00)	2.06 (1.65)	1.91 (0.85)	3.05 (1.60)	1.72 (0.72)	1.82 (1.06)
Tendon	0.67 (0.47)	2.33 (1.25)	1.13 (0.33)	1.14 (0.35)	2.00 (1.41)	1.45 (0.66)	1.08 (0.28)
Ligament	1.26 (1.31)	1.65 (1.56)	1.90 (1.60)	2.08 (1.62)	1.48 (1.43)	1.56 (1.54)	1.27 (1.12)
Bone	0.62 (1.02)	0.40 (0.58)	0.83 (1.05)	0.68 (0.79)	0.48 (0.73)	0.39 (0.49)	0.53 (0.72)
Non-Contact Injuries	2.12 (1.78)	1.50 (0.78)	1.58 (1.34)	1.50 (0.76)	2.29 (1.35)	1.35 (0.58)	1.54 (0.93)
Muscle	2.70 (2.10)	1.67 (0.62)	1.94 (1.65)	1.75 (0.77)	2.70 (1.45)	1.62 (0.72)	1.75 (1.00)
Tendon	2.00 (0.00)	2.00 (1.41)	1.00 (0.00)	1.29 (0.45)	2.20 (1.17)	1.18 (0.39)	1.09 (0.29)
Ligament	1.00 (0.00)	1.00 (0.00)	1.50 (0.50)	1.46 (0.93)	1.50 (0.71)	1.21 (0.41)	1.07 (0.26)
Bone	1.50 (0.50)	1.00 (0.00)	1.00 (0.00)	1.13 (0.33)	2.13 (1.27)	1.13 (0.33)	2.13 (1.27)
Contact Injuries	1.39 (0.59)	1.67 (1.05)	1.55 (0.74)	1.35 (0.62)	1.65 (0.90)	1.59 (0.93)	1.55 (0.76)
Muscle	1.00 (0.00)	1.33 (0.47)	1.00 (0.00)	1.00 (0.00)	1.50 (0.50)	1.33 (0.47)	0.00 (0.00)
Tendon	0.00 (0.00)	0.00 (0.00)	1.00 (0.00)	1.00 (0.00)	1.00 (0.00)	1.00 (0.00)	1.00 (0.00)
Ligament	1.67 (0.67)	1.92 (1.19)	1.77 (0.80)	1.41 (0.60)	1.95 (0.97)	1.82 (1.03)	1.60 (0.80)
Bone	1.25 (0.43)	1.00 (0.00)	1.25 (0.43)	1.40 (0.80)	1.00 (0.00)	1.00 (0.00)	1.40 (0.49)

**Table 2:** Player genotype and allele distribution of candidate variants

	*n* (%)
*COL5A1* (rs1800012)	
CC	8 (17.0)
CT	23 (48.9)
TT	16 (34.0)
C	39 (41.5)
T	55 (58.5)
*NOGGIN* (rs1372857)	
GG	9 (19.1)
AG	22 (46.8)
AA	16 (34.0)
G	40 (42.6)
A	54 (57.4)
*COL1A1* (rs1800012)	
TT	32 (69.6)
GT	14 (30.4)
GG	0 (0.0)
T	78 (84.8)
G	54 (15.2)
*ACTN3* (rs1815739)	
CC	21 (44.7)
CT	24 (51.1)
TT	2 (4.3)
C	66 (70.2)
T	28 (29.8)
*SMAD6* (rs2053423)	
CC	8 (17.0)
CT	15 (31.9)
TT	24 (51.1)
C	31 (33.0)
T	63 (67.0)
*EMILIN1* (rs2289360)	
GG	20 (42.6)
AG	15 (31.9)
AA	12 (25.5)
G	55 (58.5)
A	39 (41.5)
*CCL2* (rs2857656)	
CC	2 (4.3)
CG	24 (51.1)
GG	21 (44.7)
C	28 (29.8)
G	66 (70.2)
*IGF2* (rs3213221)	
CC	7 (14.9)
GC	24 (51.1)
GG	16 (34.0)
C	38 (40.4)
G	56 (59.6)
*COL12A1* (rs970547)	
GG	1 (2.1)
AG	13 (27.7)
AA	33 (70.2)
G	15 (16.0)
A	79 (84.0)

**Table 3:** Mean number of total muscle and tendon, ligament, and bone injuries when categorised by candidate variant genotype

Gene	Genotype	Muscle		Tendon		Ligament		Bone	
		Mean	95% CI	Mean	95% CI	Mean	95% CI	Mean	95% CI
*All injuries*									
*COL5A1*	CC	4.57	[− 0.08 to 9.22]	2.14	[− 0.70 to 4.99]	6.57	[0.90 to 12.24]	1.57	[− 0.27 to 3.41]
(rs12722)	CT	6.96	[5.15 to 8.76]	1.48	[0.77 to 2.19]	8.09	[4.25 to 1.92]	2.78	[1.56 to 4.01]
	TT	7.40	[3.76 to 11.13]	1.40	[0.54 to 2.26]	6.53	[2.37 to 10.69]	2.47	[1.36 to 3.57]
*NOGGIN*	GG	7.29	[2.67 to 11.90]	2.71	[− 0.10 to 5.53]	7.71	[1.03 to 14.40]	3.00	[1.00 to 5.00]
(rs1372857)	AG	7.73	[5.25 to 10.20]	1.27	[0.78 to 1.77]	6.73	[4.03 to 9.43]	2.82	[1.64 to 3.99]
	AA	5.13	[2.59 to 7.66]	1.44	[0.37 to 2.50]	8.00	[2.39 to 13.61]	1.81	[0.61 to 3.02]
*COL1A1*	TT	7.00	[5.27 to 8.73]	1.59	[0.92 to 2.27]	8.72	[5.51 to 11.93]	2.69	[1.77 to 3.60]
(rs1800012)	GT	6.08	2.24 to 9.92]	1.46	[0.29 to 2.63]	3.92	[2.12 to 5.73]	2.00	[0.65 to 3.35]
	GG	–	–	–	–	–	–	–	–
*ACTN3*	CC	8.15	[5.68 to 10.62]	1.95	[0.82 to 3.08]	8.45	[5.33 to 11.57]	2.85	[1.86 to 3.84]
(rs1815739)	CT	6.00	[3.82 to 8.18]	1.30	[0.76 to 1.85]	6.96	[3.04 to 10.87]	2.26	[1.05 to 3.47]
	TT	1.00	[1.00 to 1.00]	0.50	[− 5.85 to 6.85]	0.50	[− 5.85 to 6.85]	1.50	[− 4.85 to 7.85]
*SMAD6*	CC	7.57	[0.63 to 14.51]	1.57	0.39 to 2.75]	4.14	[1.30 to 6.99]	3.57	[0.70 to 6.44]
(rs2053423)	CT	6.13	[3.68 to 8.59]	2.20	[0.73 to 3.67]	11.00	[5.31 to 16.69]	3.13	[1.99 to 4.28]
	TT	6.87	[4.64 to 9.10]	1.13	[0.59 to 1.67]	5.91	[3.12 to 8.71]	1.74	[0.74 to 2.74]
*EMILIN1*	GG	6.70	[4.19 to 9.21]	1.80	[0.91 to 2.69]	8.60	[5.09 to 12.11]	2.55	[1.28 to 3.82]
(rs2289360)	AG	6.60	[4.19 to 9.01]	1.40	[0.15 to 2.65]	6.53	[1.03 to 12.04]	2.47	[1.16 to 3.77]
	AA	7.00	[2.25 to 11.75]	1.30	[0.54 to 2.06]	6.00	[2.14 to 9.86]	2.40	[0.78 to 4.02]
*CCL2*	CC	0.00	[0.00 to 0.00]	0.00	[0.00 to 0.00]	1.00	[− 11.71 to 13.71]	3.00	[3.00 to 3.00]
(rs2857656)	CG	7.48	[5.22 to 9.73]	1.96	[0.96 to 2.95]	9.17	[4.95 to 13.40]	3.00	[1.81 to 4.19]
	GG	6.55	[4.13 to 8.97]	1.25	[0.71 to 1.79]	5.85	[3.53 to 8.17]	1.85	[0.87 to 2.83]
*IGF2*	CC	5.00	[0.96 to 9.04]	0.83	[− 0.39 to 2.06]	5.33	[1.15 to 9.51]	1.17	[− 0.06 to 2.39]
(rs3213221)	GC	8.88	[6.54 to 11.21]	2.04	[1.07 to 3.02]	9.58	[5.54 to 13.62]	3.04	[2.18 to 3.90]
	GG	4.00	[1.96 to 6.04]	1.07	[0.58 to 1.56]	4.53	[1.86 to 7.20]	2.13	[0.37 to 3.90]
*COL12A1*	GG	–	–	–	–	–	–	–	–
(rs970547)	AG	7.31	[3.33 to 11.28]	1.15	[0.42 to 1.89]	9.92	[2.91 to 16.93]	3.38	[2.24 to 4.53]
	AA	6.23	[4.57 to 7.88]	1.74	[0.97 to 2.51]	6.19	[4.05 to 8.34]	2.10	[1.14 to 3.05]
*Non-Contact Injuries*									
*COL5A1*	CC	4.14	[− 0.14 to 8.43]	1.86	[− 0.73 to 4.44]	1.00	[− 0.41 to 2.41]	1.00	[− 0.41 to 2.41]
(rs12722)	CT	6.04	[4.58 to 7.50]	1.30	[0.71 to 1.89]	1.65	[0.86 to 2.44]	1.87	[0.72 to 3.02]
	TT	6.00	[2.76 to 9.24]	1.13	[0.35 to 1.91]	1.80	[0.67 to 2.93]	0.93	[0.36 to 1.51]
*NOGGIN*	GG	6.43	[2.62 to 10.24]	2.43	− 0.01 to 4.87]	1.86	[− 0.50 to 4.21]	1.86	[− 0.44 to 4.15]
(rs1372857)	AG	6.50	[4.28 to 8.72]	1.00	[0.61 to 1.39]	1.50	[0.83 to 2.17]	1.55	[0.46 to 2.63]
	AA	4.38	[2.41 to 6.34]	1.31	[0.35 to 2.28]	1.63	[0.54 to 2.71]	1.06	[0.35 to 1.78]
*COL1A1*	TT	5.91	[4.43 to 7.38]	1.38	[0.78 to 1.97]	2.06	[1.35 to 2.77]	1.59	[0.84 to 2.35]
(rs1800012)	GT	5.31	[2.01 to 8.60]	1.23	[0.21 to 2.25]	0.46	[0.06 to 0.86]	1.00	[− 0.28 to 2.28]
	GG	–	–	–	–	–	–	–	–
*ACTN3*	CC	7.20	[4.96 to 9.44]	1.55	[0.60 to 2.50]	2.10	[1.19 to 3.01]	1.90	[0.95 to 2.85]
(rs1815739)	CT	4.91	[3.24 to 6.59]	1.22	[0.66 to 1.77]	1.30	[0.56 to 2.05]	1.13	[0.19 to 2.07]
	TT	0.50	[− 5.85 to 6.85]	0.50	[− 5.85 to 6.85]	0.00	[0.00 to 0.00]	0.00	[0.00 to 0.00]
*SMAD6*	CC	7.00	0.73 to 13.27]	1.43	[0.38 to 2.48]	0.86	[0.22 to 1.50]	2.29	[− 1.04 to 5.61]
(rs2053423)	CT	5.20	[3.13 to 7.27]	1.87	[0.58 to 3.15]	2.20	[1.03 to 3.37]	1.67	[0.84 to 2.50]
	TT	5.70	[3.87 to 7.52]	0.96	[0.48 to 1.44]	1.43	[0.64 to 2.23]	1.00	[0.21 to 1.79]
*EMILIN1*	GG	5.60	[3.43 to 7.77]	1.35	[0.57 to 2.13]	2.00	[1.03 to 2.97]	1.50	[0.37 to 2.63]
(rs2289360)	AG	5.60	[3.75 to 7.45]	1.40	[0.30 to 2.50]	1.07	[0.17 to 1.97]	1.67	[0.49 to 2.84]
	AA	6.20	[1.99 to 10.41]	1.20	[0.46 to 1.94]	1.60	[0.47 to 2.73	0.90	[− 0.02 to 1.82]
*CCL2*	CC	0.00	[0.00 to 0.00]	0.00	[0.00 – 0.00]	0.00	[0.00 to 0.00]	0.50	[− 5.85 to 6.85]
(rs2857656)	CG	6.48	[4.54 to 8.41]	1.74	[0.86 to 2.62]	1.87	[0.88 to 2.86]	2.04	[0.96 to 3.13]
	GG	5.45	[3.42 to 7.48]	1.00	[0.54 to 1.46]	1.45	[0.89 to 2.01]	0.80	[0.16 to 1.44]
*IGF2*	CC	4.17	[1.02 to 7.31]	0.50	[− 0.38 to 1.38]	1.50	[0.40 to 2.60]	0.67	[− 0.60 to 1.94]
(rs3213221)	GC	7.50	[5.46 to 9.54]	1.67	[0.80 to 2.54]	2.08	[1.16 to 3.01]	1.54	[0.81 to 2.28]
	GG	3.53	[1.81 to 5.26]	1.13	[0.67 to 1.60]	0.87	[0.21 to 1.52]	1.53	[− 0.03 to 3.10]
*COL12A1*	GG	–	–	–	–	–	–	–	–
(rs970547)	AG	6.31	[2.78 to 9.83]	0.92	[0.46 to 1.38]	1.85	[0.41 to 3.28]	2.15	[0.92 to 3.38]
	AA	5.23	[3.90 to 6.55]	1.52	[0.82 to 2.21]	1.52	[0.92 to 2.11]	1.13	[0.36 to 1.90]
*Contact injuries*									
*COL5A1*	CC	0.29	[− 0.17 to 0.74]	0.29	[− 0.17 to 0.74]	5.14	[1.28 to 9.01]	0.57	[− 0.33 to 1.47]
(rs12722)	CT	0.61	[0.20 to 1.02]	0.13	[− 0.02 to 0.28]	4.61	[3.00 to 6.22]	0.65	[0.23 to 1.08]
	TT	0.33	[− 0.01 to 0.68]	0.20	[− 0.03 to 0.43]	4.07	[1.29 to 6.85]	1.27	[0.47 to 2.06]
*NOGGIN*	GG	0.43	[− 0.07 to 0.92]	0.29	[− 0.17 to 0.74]	5.29	[1.48 to 9.09]	1.14	[0.02 to 2.27]
(rs1372857)	AG	0.55	[0.25 to 0.84]	0.23	[0.04 to 0.42]	4.32	[2.49 to 6.15]	0.91	[0.46 to 1.36]
	AA	0.38	[− 0.17 to 0.92]	0.06	[− 0.07 to 0.20]	4.44	[2.04 to 6.84]	0.65	[− 0.10 to 1.35]
*COL1A1*	TT	0.59	[0.28 to 0.91]	0.22	[0.07 to 0.37]	5.09	[3.42 to 6.77]	0.94	[0.49 to 1.39]
(rs1800012)	GT	0.15	[− 0.07 to 0.38]	0.08	[− 0.09 to 0.24]	3.08	[1.67 to 4.48]	0.62	[0.03 to 1.20]
	GG	–	–	–	–	–	–	–	–
*ACTN3*	CC	0.45	[0.13 to 0.77]	0.25	[0.04 to 0.46]	5.45	[3.43 to 7.47]	0.80	[0.35 to 1.25]
(rs1815739)	CT	0.52	[0.13 to 0.91]	0.13	[− 0.02 to 0.28]	4.09	[2.35 to 5.82]	0.87	[0.27 to 1.47]
	TT	0.00	[0.00 to 0.00]	0.00	[0.00 to 0.00]	0.00	[0.00 to 0.00]	1.00	[1.00 to 1.00]
*SMAD6*	CC	0.29	[− 0.17 to 0.74]	0.00	[0.00 to 0.00]	2.86	[0.69 to 5.02]	0.86	[− 0.27 to 1.98]
(rs2053423)	CT	0.53	[0.18 to 0.89]	0.27	[0.01 to 0.52]	6.20	[3.77 to 8.63]	1.13	[0.51 to 1.76]
	TT	0.48	[0.07 to 0.89]	0.17	[0.01 to 0.34]	3.91	[2.08 to 5.75]	0.65	[0.14 to 1.17]
*EMILIN1*	GG	0.50	[0.18 to 0.82]	030	[0.08 to 0.52]	5.40	[3.15 to 7.65]	0.85	[0.26 to 1.44]
(rs2289360)	AG	0.33	[0.06 to 0.60]	0.07	[− 0.08 to 0.21]	3.80	[1.88 to 5.72]	0.60	[0.14 to 1.06]
	AA	0.60	[− 0.30 to 1.50]	0.10	[− 0.13 to 0.33]	3.80	[1.04 to 6.56]	1.20	[0.20 to 2.20]
*CCL2*	CC	0.00	[0.00 to 0.00]	0.00	[0.00 – 0.00]	0.50	[− 5.85 to 6.85]	1.00	[− 11.71 to 13.71]
(rs2857656)	CG	0.48	[0.09 to 0.87]	0.17	[0.01 to 0.34]	5.52	[3.51 to 7.53]	0.78	[0.26 to 1.30]
	GG	0.50	[0.18 to 0.82]	0.20	[0.01 to 0.39]	3.75	[2.14 to 5.36]	0.90	[0.35 to 1.45]
*IGF2*	CC	0.50	[− 0.38 to 1.38]	0.33	[− 0.21 to 0.88]	3.50	[0.55 to 6.45]	0.50	[− 0.38 to 1.38]
(rs3213221)	GC	0.63	[0.23 to 1.02]	0.25	[0.06 to 0.44]	5.67	[3.71 to 7.62]	1.29	[0.76 to 1.83]
	GG	0.20	[− 0.03 to 0.43]	0.00	[0.00 to 0.00]	3.07	[1.15 to 4.98]	0.27	[− 0.18 to 0.71]
*COL12A1*	GG	–	–	–	–	–	–	–	–
(rs970547)	AG	0.46	[0.06 to 0.86]	0.08	[− 0.09 to 0.24]	5.54	[2.53 to 8.55]	0.85	[0.25 to 1.44]
	AA	0.45	[0.14 to 0.76]	0.23	[0.07 to 0.38]	4.00	[2.59 to 5.41]	0.81	[0.35 to 1.26]

Genetic associations with the total number of injuries, with muscle, tendon, ligament, and bone are presented in Table 4. The rs1372857 variant within *NOGGIN* (*p* = 0.050) and the rs12722 variant within the *COL5A1* (*p* = 0.028) genes were the variants associated with all muscle-related injuries. Trends were seen for the rs2857656 *CCL2* (*p* = 0.082) and the rs3213221 *IGF2* (*p* = 0.097) variants. Trends were also seen with *COL1A1* rs1800012 for non-contact muscle injuries (*p* = 0.100) and *NOGGIN* rs1372857 for contact muscle injuries (*p* = 0.054). Associations were seen for the *NOGGIN* rs1372857 variant for moderate (*p* = 0.044) and moderate and high combined (*p* = 0.016) severity total muscle injuries. Trends were seen between *NOGGIN* rs1372857 and high severity total muscle injuries (*p* = 0.084), as well as for the *COL5A1* rs12722 variant for low (*p* = 0.073) and moderate (*p* = 0.073) severity total muscle injuries. There is an association for the rs3213221 *IGF2* variant for contact tendon injuries (p = 0.028). Trends were seen for the rs970547 *COL12A1* variant for all tendon (*p* = 0.084) and non-contact tendon injuries (*p* = 0.079); however, an association for the rs970547 *COL12A1* variant was seen for low severity contact tendon injuries (*p* = 0.026). Significant associations were found for ligament injuries, specifically between the rs1800012 *COL1A1* variant and all ligament injuries (*p* = 0.019), with further associations observed with low severity (*p* = 0.002), and non-contact ligament injuries (*p* = 0.002), with further associations with low severity rated non-contact injuries (*p* = 0.004). The rs12722 *COL5A1* variant was associated with contact bone injuries (*p* = 0.030), with a further association seen with the number of moderate severity injuries (*p* = 0.049). A trend was also seen with respect to the number of moderate and high combined severity injuries (*p* = 0.065).

**Table 4:** Negative binominal model of genetic variants and total number of muscle, tendon, ligament, and bone-related injuries

Variant		Muscle			Tendon			Ligament			Bone		
		IRR	p value	95% CI	IRR	p value	95% CI	IRR	p value	95% CI	IRR	p value	95% CI
*All injuries*													
*COL5A1*	Intercept	0.026	0.000	[0.02 to 0.04]	0.012	0.000	[0.00 to 0.03]	0.037	0.000	[0.02 to 0.08]	0.10	0.000	[0.00 to 0.02]
(rs12722)	*COL5A1*	1.253	**0.028**	[1.03 to 1.53]	0.920	0.705	[0.60 to 1.42]	1.114	0.504	[0.81 to 1.53]	1.206	0.244	[0.88 to 1.65]
*NOGGIN*	Intercept	0.066	0.000	[0.04 to 0.11]	0.016	0.000	[0.01 to 0.04]	0.043	< 0.001	[0.02 to 0.10]	0.024	< 0.001	[0.01 to 0.04]
(rs1372857)	*NOGGIN*	0.813	**0.050**	[0.66 to 1.00]	0.802	0.285	[0.54 to 1.20]	1.038	0.833	[0.74 to 1.46]	0.820	0.159	[0.60 to 1.09]
*COL1A1*	Intercept	0.044	0.000	[0.03 to 0.07]	0.009	0.000	[0.00 to 0.02]	0.090	< 0.001	[0.05 to 0.17]	0.021	0.000	[0.01 to 0.04]
(rs1800012)	*COL1A1*	0.967	0.842	[0.69 to 1.34]	1.087	0.808	[0.55 to 2.13]	0.567	**0.019**	[0.35 to 0.91]	0.763	0.360	[0.43 to 1.36]
*ACTN3*	Intercept	0.055	0.000	[0.04 to 0.09]	0.012	0.000	[0.01 to 0.03]	0.045	0.000	[0.02 to 0.08]	0.016	0.000	[0.01 to 0.03]
(rs1815739)	*ACTN3*	0.845	0.226	[0.64 to 1.11]	0.861	0.594	[0.50 to 1.49]	1.031	0.884	[0.69 to 1.54]	0.972	0.896	[0.63 to 1.50]
*SMAD6*	Intercept	0.034	0.000	[0.02 to 0.05]	0.011	0.000	[0.00 to 0.03]	0.040	0.000	[0.02 to 0.08]	0.022	0.000	[0.01 to 0.04]
(rs2053423)	*SMAD6*	1.103	0.317	[0.91 to 1.34]	0.938	0.758	[0.62 to 1.41]	1.074	0.590	[0.83 to 1.40]	0.844	0.207	[0.65 to 1.10]
*EMILIN1*	Intercept	0.040	0.000	[0.03 to 0.06]	0.014	0.000	[0.01 to 0.03]	0.063	0.000	[0.04 to 0.10]	0.018	0.000	[0.01 to 0.03]
(rs2289360)	*EMILIN1*	1.040	0.672	[0.88 to 1.25]	0.824	0.320	[0.56 to 1.21]	0.826	0.141	[0.64 to 1.07]	0.905	0.506	[0.67 to 1.21]
*CCL2*	Intercept	0.023	0.000	[0.01 to 0.05]	0.013	< 0.001	[0.00 to 0.05]	0.091	< 0.001	[0.03 to 0.25]	0.042	< 0.001	[0.01 to 0.13]
(rs2857656)	*CCL2*	1.292	0.082	[1.00 to 1.23]	0.887	0.679	[0.50 to 1.56]	0.756	0.208	[0.49 to 1.17]	0.646	0.093	[0.39 to 1.08]
*IGF2*	Intercept	0.066	0.000	[0.04 to 0.11]	0.010	< 0.001	[0.00 to 0.03]	0.076	< 0.001	[0.03 to 0.18]	0.007	0.000	[0.00 to 0.02]
(rs3213221)	*IGF2*	0.814	0.097	[0.64 to 1.04]	0.996	0.988	[0.59 to 1.69]	0.799	0.277	[0.53 to 1.20]	1.393	0.093	[0.95 to 2.05]
*COL12A1*	Intercept	0.051	0.000	[0.03 to 0.10]	0.002	< 0.001	[0.00 to 0.01]	0.080	< 0.001	[0.04 to 0.17]	0.019	0.000	[0.00 to 0.05]
(rs970547)	*COL12A1*	0.937	0.623	[0.72 to 1.22]	1.698	0.084	[0.93 to 3.10]	0.812	0.181	[0.60 to 1.10]	0.920	0.639	[0.65 to 1.30]
*Non-contact injuries*													
*COL5A1*	Intercept	0.025	0.000	[0.02 to 0.04]	0.011	0.000	[0.00 to 0.03]	0.005	0.000	[0.00 to 0.01]	0.11	< 0.001	[0.00 to 0.05]
(rs12722)	*COL5A1*	1.188	0.120	[0.96 to 1.48]	0.882	0.582	[0.57 to 1.38]	1.384	0.127	[0.91 to 2.10]	0.919	0.802	[0.48 to 1.78]
*NOGGIN*	Intercept	0.057	0.000	[0.04 to 0.09]	0.013	0.000	[0.00 to 0.03]	0.008	0.000	[0.00 to 0.02]	0.013	< 0.001	[0.00 to 0.05]
(rs1372857)	*NOGGIN*	0.810	0.054	[0.66 to 1.00]	0.811	0.324	[0.54 to 1.23]	1.081	0.712	[0.71 to 1.64]	0.837	0.549	[0.47 to 1.50]
*COL1A1*	Intercept	0.036	0.000	[0.02 to 0.06]	0.008	0.000	[0.00 to 0.02]	0.049	< 0.001	[0.02 to 0.13]	0.014	< 0.001	[0.00 to 0.05]
(rs1800012)	*COL1A1*	1.009	0.961	[0.72 to 1.42]	1.042	0.906	[0.52 to 2.08]	0.255	**0.002**	[0.11 to 0.60]	0.711	0.476	[0.28 to 1.82]
*ACTN3*	Intercept	0.051	0.000	[0.03 to 0.08]	0.009	0.000	[0.00 to 0.02]	0.015	0.000	[0.01 to 0.04]	0.014	< 0.001	[0.00 to 0.05]
(rs1815739)	*ACTN3*	0.795	0.110	[0.60 to 1.05]	0.991	0.974	[0.57 to 1.73]	0.745	0.275	[0.44 to 1.26]	0.746	0.448	[0.35 to 1.59]
*SMAD6*	Intercept	0.032	0.000	[0.02 to 0.05]	0.010	0.000	[0.00 to 0.03]	0.007	0.000	[0.00 to 0.02]	0.020	< 0.001	[0.01 to 0.07]
(rs2053423)	*SMAD6*	1.057	0.588	[0.87 to 1.29]	0.907	0.644	[0.60 to 1.37]	1.191	0.388	[0.80 to 1.77]	0.704	0.204	[0.41 to 1.21]
*EMILIN1*	Intercept	0.032	0.000	[0.02 to 0.05]	0.009	0.000	[0.00 to 0.02]	0.014	0.000	[0.01 to 0.03]	0.011	0.000	[0.00 to 0.03]
(rs2289360)	*EMILIN1*	1.069	0.483	[0.89 to 1.29]	0.950	0.798	[0.64 to 1.40]	0.834	0.313	[0.59 to 1.19]	0.897	0.693	[0.53 to 1.54]
*CCL2*	Intercept	0.022	0.000	[0.01 to 0.05]	0.014	< 0.001	[0.00 to 0.05]	0.007	< 0.001	[0.00 to 0.03]	0.031	< 0.001	[0.01 to 0.19]
(rs2857656)	*CCL2*	1.236	0.170	[0.91 to 1.67]	0.812	0.480	[0.46 to 1.45]	1.128	0.669	[0.65 to 1.96]	0.586	0.160	[0.28 to 1.24]
*IGF2*	Intercept	0.053	0.000	[0.03 to 0.09]	0.005	< 0.001	[0.00 to 0.02]	0.025	< 0.001	[0.01 to 0.07]	0.008	< 0.001	[0.00 to 0.04]
(rs3213221)	*IGF2*	0.838	0.175	[0.65 to 1.08]	1.272	0.389	[0.74 to 2.20]	0.648	0.082	[0.40 to 1.06]	1.071	0.846	[0.54 to 2.14]
*COL12A1*	Intercept	0.049	0.000	[0.02 to 0.10]	0.002	< 0.001	[0.00 to 0.01]	0.008	< 0.001	[0.00 to 0.03]	0.041	0.005	[0.00 to 0.37]
(rs970547)	*COL12A1*	0.894	0.405	[0.69 to 1.16]	1.750	0.079	[0.94 to 2.37]	1.085	0.765	[0.64 to 1.86]	0.561	0.170	[0.25 to 1.28]
*Contact injuries*													
*COL5A1*	Intercept	0.003	< 0.001	[0.00 to 0.01]	0.001	< 0.001	[0.00 to 0.01]	0.035	0.000	[0.02 to 0.07]	0.001	0.000	[0.00 to 0.01]
(rs12722)	*COL5A1*	1.021	0.950	[0.53 to 1.97]	0.979	0.967	[0.35 to 2.76]	0.906	0.518	[0.67 to 1.22]	1.824	**0.030**	[1.06 to 3.13]
*NOGGIN*	Intercept	0.003	< 0.001	[0.00 to 0.01]	0.004	< 0.001	[0.01 to 0.03]	0.028	0.000	[0.02 to 0.05]	0.009	0.000	[0.00 to 0.03]
(rs1372857)	*NOGGIN*	0.930	0.824	[0.49 to 1.76]	0.528	0.216	[0.19 to 1.45]	0.998	0.989	[0.75 to 1.33]	0.768	0.288	[0.47 to 1.25]
*COL1A1*	Intercept	0.012	< 0.001	[0.00 to 0.06]	0.003	< 0.001	[0.00 to 0.04]	0.042	0.000	[0.02 to 0.08]	0.008	< 0.001	[0.00 to 0.03]
(rs1800012)	*COL1A1*	0.294	0.100	[0.07 to 1.26]	0.399	0.390	[0.05 to 3.24]	0.727	0.184	[0.46 to 1.16]	0.736	0.502	[0.30 to 1.80]
*ACTN3*	Intercept	0.002	0.000	[0.00 to 0.01]	0.002	< 0.001	[0.00 to 0.02]	0.037	0.000	[0.02 to 0.07]	0.003	0.000	[0.00 to 0.01]
(rs1815739)	*ACTN3*	1.273	0.545	[0.58 to 2.79]	0.638	0.521	[0.16 to 2.52]	0.840	0.364	[0.58 to 1.22]	1.552	0.151	[0.85 to 2.83]
*SMAD6*	Intercept	0.001	< 0.001	[0.00 to 0.01]	< 0.001	< 0.001	[0.00 to 0.01]	0.021	0.000	[0.01 to 0.04]	–	–	–
(rs2053423)	*SMAD6*	1.341	0.353	[0.72 to 2.49]	1.567	0.400	[0.55 to 4.46]	1.130	0.399	[0.85 to 1.50]	–	–	–
*EMILIN1*	Intercept	0.003	0.000	[0.00 to 0.01]	0.004	< 0.001	[0.00 to 0.02]	0.039	0.000	[0.02 to 0.06]	0.004	0.000	[0.00 to 0.01]
(rs2289360)	*EMILIN1*	1.040	0.889	[0.60 to 1.81]	0.447	0.163	[0.14 to 1.39]	0.830	0.143	[0.65 to 1.07]	1.175	0.491	[0.74 to 1.86]
*CCL2*	Intercept	0.001	< 0.001	[0.00 to 0.01]	< 0.001	< 0.001	[0.00 to 0.01]	0.031	< 0.001	[0.01 to 0.08]	0.005	< 0.001	[0.00 to 0.03]
(rs2857656)	*CCL2*	1.349	0.477	[0.59 to 3.08]	1.469	0.572	[0.39 to 5.58]	0.963	0.845	[0.66 to 1.41]	1.066	0.858	[0.53 to 2.13]
*IGF2*	Intercept	0.010	< 0.001	[0.00 to 0.05]	0.017	< 0.001	[0.00 to 0.17]	0.039	0.000	[0.02 to 0.08]	0.015	< 0.001	[0.00 to 0.06]
(rs3213221)	*IGF2*	0.559	0.146	[0.26 to 1.23]	0.249	**0.028**	[0.07 to 0.86]	0.865	0.392	[0.62 to 1.21]	0.603	0.108	[0.33 to 1.12]
*COL12A1*	Intercept	0.003	< 0.001	[0.00 to 0.02]	< 0.001	< 0.001	[0.00 to 0.01]	0.038	< 0.001	[0.01 to 0.10]	0.006	< 0.001	[0.00 to 0.03]
(rs970547)	*COL12A1*	0.987	0.974	[0.46 to 2.14]	3.506	0.225	[0.46 to 26.62]	0.896	0.562	[0.62 to 1.30]	0.946	0.856	[0.52 to 1.72]

Significant effects are **bolded**

The *NOGGIN* rs1372857 variant had an IRR of 0.813 [0.66 to 1.00] for total muscle injuries, with analysis finding those with the *NOGGIN* GG genotype experiencing an estimated number of injuries per game of 8.44 [6.16 to 1.71], compared to 6.86 [7.27 to 9.40] for the heterozygous genotype and 5.58 [4.28 to 6.87] for the AA genotype. When examining moderate severity injuries, the IRR was 0.691 [0.48 to 0.99], with the estimated number of injuries being 3.45 (GG) [1.88 to 5.01], 2.38 (AG) [1.80 to 2.95], and 1.64 (AA) [0.96 to 2.32]. When analysing moderate and high severity injuries, the IRR was 0.639 [0.44 to 0.92], with the estimated number of injuries being 4.71 (GG) [2.53 to 6.89], 3.01 (AG) [2.27 to 3.74], and 1.92 (AA) [1.13 to 2.72].

The *COL5A1* rs12722 variant had an IRR of 1.253 [1.03 to 1.53] for total muscle injuries, with the TT genotype having a higher estimated number of injuries per game (8.00 [6.37 to 9.61]) than the CT (6.38 [5.50 to 7.25]) and CC genotypes (5.09 [3.67 to 6.51]).

In terms of contact tendon injuries, the *IGF2* rs3213221 variant had an IRR of 0.249 [0.07 to 0.86], with the CC genotype (0.66 [− 0.11 to 1.44] having a higher estimated number of injuries per games than the CG (0.17 [0.04 to 0.29]) and GG (0.04 [− 0.03 to 0.11]) genotypes. When investigating low severity contact tendon injuries, the IRR was 0.222 [0.06 to 0.84], with the CC genotype (0.63 [− 0.13 to 1.40] having a higher estimated number of injuries per games than the CG (0.14 [0.02 to 0.26]) and GG (0.03 [− 0.03 to 0.09]) genotypes.

For the *COL1A1* rs1800012 variant, with an observed IRR of 0.567 [0.35 to 0.91] for total ligament injuries, carriers of the TT genotype experienced an estimated number of injures per game of 8.12 [6.31 to 9.92], compared to 4.61 [2.69 to 6.52] for the heterozygous genotype. The GG genotype was not represented in the current population. The IRR for low severity injuries was 0.602 [0.38 to 0.94], with the estimated number of injuries of the TT and GT genotypes being 7.00 [5.61 to 8.40] and 4.21 [2.52 to 5.91], respectively. For non-contact ligament injuries, the rs1800012 variant had an IRR of 0.255 [0.11 to 0.60], with the TT genotype having a higher estimated number of injuries per game (1.99 [1.48 to 2.50]) compared to the GT genotype (0.51 [0.10 to 0.92]). When analysing low severity non-contact ligament injuries, the IRR was 0.243, with the TT genotype (1.75 [1.22 to 2.27]) having a higher number of estimated injuries per game than the GT genotype (0.42 [0.04 to 0.81]).

For contact bone injuries, the *COL5A1* rs12722 variant had an IRR of 1.824 [1.06 to 3.13] with the TT genotype (1.28 [0.66 to 1.90]) having a higher number of estimated injuries per game compared to the CT (0.70 [0.42 to 0.98]) and CC genotypes (0.38 [0.07 to 0.70]). There were significant associations for moderate contact bone injuries with an IRR of 2.82 [1.01 to 1.89] with TT genotype (0.47 [0.12 to 0.82]) having a higher number of estimated injuries per game compared to the CT (0.17 [0.03 to 0.30]) and CC genotypes (0.06 [− 0.04 to 0.16]).

## Discussion

Our preliminary investigation into the association of candidate genetic variants with injury number and severity in elite AFL players resulted in novel findings, and the identification of novel genetic markers for injury classification within AF. We discovered an association between the *NOGGIN* polymorphism (rs1372857) and all muscle injuries, with significantly higher muscle injury incidence for those with the GG genotype, which also trended towards moderate-to-high combined severity injuries. The rs1372857 variant within the *NOGGIN* gene has been linked to bone fractures in a clinical study investigating motor vehicle accident, fall, and direct blow patients [31], with the homozygous GG genotype associated with non-union fractures [31]. While we did not identify a relationship between rs1372857 and bone injuries in our AFL cohort, we did observe a similar link between the GG genotype and a larger number of total injuries per season, together with more moderate-to-high severity of injuries. Given the inextricable link between muscle, tendon, and bone, and their co-adaptive processes [9], it seems reasonable to observe some crossover with *NOGGIN* expression and injuries to these tissues. Further research needs to be done to confirm whether this association between genetic variants within *NOGGIN* and subsequent muscle and tendon injuries in AFL players exists.

The current study found an association between the *COL5A1* rs12722 polymorphism and all muscle injuries, with the TT genotype having a higher estimated number of injuries per game. This follows previously reported associations between the CC genotype of rs12722 and less severe non-contact muscle injuries in soccer players [26, 55]. However, the variant was also found to show no significant difference between muscle injury and no muscle injury groups in a Japanese group of varied athletes [44]. The *COL5A1* gene encodes for the collagen type V α1 chain of protein and forms part of the extracellular matrix of skeletal muscles and can affect passive muscle stiffness as well as joint flexibility [56, 57]. The rs12722 polymorphism has also been associated with a higher susceptibility to ligament injuries [58]. We also found significant associations between the rs12722 TT genotype, contact bone injuries, and moderate severity contact bone injuries. Due to the findings being with contact, the polymorphism may have more of an effect on response to acute trauma.

We also found an association with the *IGF2* rs3213221 polymorphism and contact tendon injuries. This variant has previously been linked with tendon and muscle injuries in elite Caucasian soccer players, with a higher number of tendon injuries related to the presence of the C allele [59]. Our study found that those with the CC genotype had a higher estimated number of injuries per game compared to its counterparts, which coincides with previous findings. *IGF2* plays a role in modulating satellite cell activation and differentiation, thereby affecting soft tissue growth, as well as response to cell degeneration and regeneration following injury [26, 60].

Our study also discovered a significant association between the total number of ligament injuries with the rs1800012 variant of the *COL1A1* gene, with a significantly higher likelihood of low severity ligament-related injuries in those with the *COL1A1* TT genotype, and a significant association between non-contact ligament injuries. These results are contradictory to previous research that has found that those with the TT genotype had less prevalence of ACL injuries [61] and cruciate ligament or shoulder dislocation injuries [62]. The *COL1A1* gene encodes the protein chain in type 1 collagen, a structural component in ligaments [61]. The T allele of the *COL1A1* gene is associated with an increased production the protein chain in type 1 collagen [62]. Previous studies have investigated the effect of collagen peptides in muscle damage post-eccentric training [63] and in post-traumatic osteoarthritis in mice [64] and suggest that the supplementation of collagen may reduce inflammation. It has also been suggested that the increased production of the protein chain in type 1 collagen associated with the T allele increases the tensile strength of tendons and ligaments; however, the precise mechanism is unknown [62]. The contradictory results of the current study could be due to the GG genotype not being represented in the current population. Similarly, ligament injuries are multifactorial, ligaments are passive anatomical structures, and injuries cannot be solely predicted by genetic variations alone; thus, it could be the significant association between the *COL1A1* TT genotype and low-grade ligament injuries which infers a possible protective effect resulting in lower-grade rather than higher-grade injuries when the injurious events occur. This is speculative and would require thorough exploration of these associations and implications between *COL1A1* and the TT genotype with ligament injuries in AFL more broadly.

Stress fractures are prevalent in AF, particularly for first- and second-year players who have less training and game experience at the elite level, and immature or developing physical structures yet to wholly tolerate these elevated physical demands [7, 11, 49, 65]. Although our study found no significant result between bone injuries and the genetic variants we explored in our AFL cohort, this may be due to the prospective observation of the same forty-six of various physical maturity overtime. Instead, for bone stress injuries (as disparate from other injury types), it may be better for future studies to prospectively observe early career AFL players in their first two seasons [66], who are typical candidates for stress fractures [65, 67], and use these early career cohorts to delineate genetic variant differences between those who sustain or avoid stress fractures. Despite previous associations with bone mineral density, the variant within the *SMAD6* gene had no significant association within the current study.

This study has several strengths and limitations that warrant acknowledgement. This is the first study to explore the associations between a select panel of genetic variants to different types of injuries in elite AFL players. Another strength was the collection of injury incidence and severity data over 7 consecutive years using the AFL’s highly standardised and reliable injury recording and reporting methods. However, our study’s limitations include its small sample size for a genetic study, and the single elite AFL squad investigated, ensuring the results must be delimited to this one cohort. Age represents one important contributor to injury risk though it was unable to be evaluated in our statistical model. In addition, the panel of genes included in this study will not be reflective of all genetic variants that may be important, and thus, our results are also delimited to the gene candidates evaluated. Replication of the current study using as many elite AFL teams as possible (i.e. preferably competition-wide studies), and broader range of genetic variants of interest, would be recommended to maximise this line of investigation and seek to confirm our findings into elite-level male footballers and the genetic underpinnings of injury incidence and severity. Lastly, predisposition to, or associations with, injuries is likely to be highly complex and polygenic in nature; thus, future research could focus on the cumulative impact of genetic variation assessed through polygenic risk scores, such as the total genotype score (TGS) method [68]. Regardless, our preliminary study raises some intriguing insights into potential relationships among genotypes and its link to injury incidence and severity that warrants further investigation.

## Conclusion

Several novel and significant associations were found during the current study. The rs12722 SNP within the *COL5A1* gene was significantly associated with all muscle injuries, as was *NOGGIN* rs1372857, while *COL5A1* was also significantly associated with contact bone injuries. The *IGF2* rs3213221 polymorphism was significantly associated with contact tendon injuries, while the *COL1A1* rs1800012 gene was significantly associated with all ligament injuries, with further associations with low severity injuries and non-contact ligament injuries. Several trends towards significance were observed between *CCL2* rs2857656 and *IGF2* rs3213221 for all muscle injuries, between *COL1A1* rs1800012 and contact muscle injuries, between *NOGGIN* rs1372857 and non-contact muscle injuries, and *COL12A1* rs970547 and all tendon and non-contact tendon injuries. Future research should expand the population pool by undertaking a competition-wide study and may investigate how such genetic variants could influence a person’s injury rate when compared to playing position, physiological abilities, and training loads or exposure time (hours of training and competition) across a season. Future research should replicate this work in elite female Australian Football (AFLW) population.

These findings present potential applications for developing training regimes around genetic predisposition. Players who are known to have a higher risk of injury due to underlying genetic susceptibilities could have more specific training based around their individual needs, including targeted and specific strength programs, and more scrutinised loading practises to reduce injury onset. For example, elite male Australian Football players with genotypes associated with potential susceptibility to bone-related injuries should be prioritised for (1) closer screening and ongoing monitoring of lower-body musculoskeletal morphology status and training adaptation through dual-energy X-ray absorptiometry (DXA) and peripheral quantitative computed tomography (pQCT) if available; (2) individualised load management practices with lower running volumes (relative to others) in favour of targeted lower-body mechanical loading programs honouring osteogenic principles of mechano-adaptation (i.e. high-magnitude, low-volume strength training, or multi-directional plyometric exercises) to optimise musculoskeletal cross-sectional area, promote skeletal robustness, and improve skeletal fatigue resistance [69–72]; and (3) nutritional review by a sports dietitian to evaluate energy availability, calcium, and vitamin D intake for potential supplementation in accordance with the Australian Football Anti-Doping Code (signatory to the World Anti-Doping Code, World Anti-Doping Agency (WADA)) [73–75].

## Data Availability

Raw datasets generated and analysed during this study are not publicly available due to agreement with the football club, and to protect the confidentiality and individual privacy of the athlete participants within the elite football club.

## Abbreviations

ACL: Anterior Cruciate Ligament
ACTN3: Alpha-Actinin-3
AF: Australian Football
AFL: Australian Football League
AFLW: Australian Football League Women
AGRF: Australian Genome Research Facility
BMP: Bone Morphogenetic Proteins
CCL2: Chemokine CC Motif Ligand-2
COL1A1: Collagen Type I Alpha 1
COL12A1: Collagen Type XII Alpha 1
COL5A1: Collagen Type V Alpha 1
DNA: Deoxyribonucleic Acid
DXA: Dual-energy X-ray Absorptiometry
EMILIN1: Elastin Microfibril Interface 1
HWE: Hardy–Weinberg Equilibrium
IGF2: Insulin-like Growth Factor-2
IRRs: Incident Rate Ratios
pQCT: Peripheral Quantitative Computed Tomography
SMAD6: SMAD Family Member 6
SNP: Single Nucleotide Polymorphism
TGS: Total Genotype Score
WADA: World Anti-Doping Agency
WAFL: Western Australian Football League
